# Trapezium Bone Density—A Comparison of Measurements by DXA and CT

**DOI:** 10.3390/jfb9010009

**Published:** 2018-01-18

**Authors:** Sebastian Breddam Mosegaard, Kamille Breddam Mosegaard, Nadia Bouteldja, Torben Bæk Hansen, Maiken Stilling

**Affiliations:** 1 University Clinic for Hand, Hip and Knee Surgery, Regional Hospital Holstebro, Hospital Unit West, 7500 Holstebro, Denmark; kamille.breddam@gmail.com (K.B.M.); torbehns@rm.dk (T.B.H.); maiken.stilling@clin.au.dk (M.S.); 2 Department of Clinical Medicine, University of Aarhus, 8000 Aarhus, Denmark; 3 Department of Radiology, Regional Hospital Holstebro, Hospital Unit West, 7500 Holstebro, Denmark; nadibout@rm.dk

**Keywords:** trapezium, bone mineral density, Hounsfield units, TM joint osteoarthritis, bone quality

## Abstract

Bone density may influence the primary fixation of cementless implants, and poor bone density may increase the risk of implant failure. Before deciding on using total joint replacement as treatment in osteoarthritis of the trapeziometacarpal joint, it is valuable to determine the trapezium bone density. The aim of this study was to: (1) determine the correlation between measurements of bone mineral density of the trapezium obtained by dual-energy X-ray absorptiometry (DXA) scans by a circumference method and a new inner-ellipse method; and (2) to compare those to measurements of bone density obtained by computerized tomography (CT)-scans in Hounsfield units (HU). We included 71 hands from 59 patients with a mean age of 59 years (43–77). All patients had Eaton–Glickel stage II–IV trapeziometacarpal (TM) joint osteoarthritis, were under evaluation for trapeziometacarpal total joint replacement, and underwent DXA and CT wrist scans. There was an excellent correlation (r = 0.94) between DXA bone mineral density measures using the circumference and the inner-ellipse method. There was a moderate correlation between bone density measures obtained by DXA- and CT-scans with (r = 0.49) for the circumference method, and (r = 0.55) for the inner-ellipse method. DXA may be used in pre-operative evaluation of the trapezium bone quality, and the simpler DXA inner-ellipse measurement method can replace the DXA circumference method in estimation of bone density of the trapezium.

## 1. Introduction

Implant failure in total joint replacement of the trapeziometacarpal joint is a major problem, especially concerning the trapezium cup component [1,2,3,4,5,6]. The reasons are unclear, and implant failures may be caused by multiple independent factors such as implant design, insertion technique, and patient-related factors [7]. Impaired bone quality of the trapezium may represent a failure risk especially in cementless implants, but the relation between hand osteoarthritis and osteoporosis is unclear [8,9,10,11,12,13,14]. Assessment of bone quality is not straightforward, as it is influenced by factors including the bone mineral, bone collagen, and microscopic architecture of the bone [15]. Predicting poor bone density of the trapezium before surgery may therefore guide the choice of surgery towards non-prosthetic treatment and potentially reduce the failure rate of TM total joint implants. Hansen et al. [16] tested the precision and reliability of measurements of trapezium bone mineral density (BMD) in patients with osteoarthritic TM joints using dual-energy X-ray absorptiometry scans (DXA-scans), and found that it is possible to measure the BMD of the trapezium with sufficient precision for clinical use. However, identification of the borders of the trapezium is difficult and time-consuming, although precision has been shown to be acceptable [16]. DXA scans may not always be available, and therefore alternative measurement methods should be considered. Hounsfield units are a standardized computed tomography (CT) attenuation coefficient. Schreiber et al. [17] tested if Hounsfield units on CT scans correlated with BMD in a clinical study with patients undergoing both lumbar spine DXA and CT imaging. They found moderate correlations between both BMD (r^2^ = 0.44) and T-scores (r^2^ = 0.48) using DXA scans and Hounsfield units, and concluded that relevant clinical CT scans performed for other purposes can provide an alternative method for determining regional bone density at no additional expense for the patient. If the correlation between DXA and CT measured bone mineral density of the trapezium is good, the preoperative CT scans of the wrist may be used to determine the trapezium bone density prior to decision making of surgical treatment in TM joint osteoarthritis.

The aim of this study was to: (1) compare the DXA bone mineral density measurements of the trapezium using a previously described method to mark the circumference of the trapezium in comparison with a new and simpler method to mark the inner-ellipse of the trapezium; and (2) determine whether these DXA measurements correlate with Hounsfield units obtained from CT scans of the trapeziums.

## 2. Results

Our BMD measurements did not differ from the reference material in any of the three age groups (Table 1). We found higher trapezium BMD for men using DXA circumference method (*p* < 0.001) and by using DXA inner-ellipse method (*p* = 0.01). We did not find a difference in CT-measured HU between males and females (*p* = 0.42) (Table 2).

We found no significant difference in CT-measured HU between age groups (*p* = 0.09), neither in DXA circumference measured BMD (*p* = 0.19) or in DXA inner-ellipse measured BMD (*p* = 0.17) between age groups (Table 3).

This was further analyzed using multiple regression analysis, showing no effects of sex and age on CT-measured Hounsfield units (HU), or any effect of age on DXA inner-ellipse measured BMD or on DXA circumference measured BMD (Table 4). The analysis showed higher BMD for men measured by the DXA inner-ellipse method (*p* = 0.004) and DXA circumference method (*p* = 0.002) (Table 4).

We found excellent repeatability for intra-observer repeated measurements with DXA inner-ellipse method and CT (Table 5).

We found an excellent correlation (r = 0.94 (95% CI: 0.90–0.96), *p* < 0.001) between the DXA circumference measured BMD of the trapezium and the DXA inner-ellipse measured BMD of the trapezium. In the trapezium there was a moderate correlation between the CT-measured HU and the DXA-measured BMD using the circumference method (r = 0.49 (95% CI: 0.29–0.65), *p* < 0.001), and with the DXA-measured BMD using the inner-ellipse method (r = 0.55 (95% CI: 0.36–0.69), *p* < 0.001). In the distal radius we also found a moderate/good correlation (r = 0.67 (95% CI: 0.52–0.78), *p* < 0.001) between the CT-measured HU and the BMD measured by DXA. We did not find a statistically significant difference between the correlations of the CT-measured HU and the circumference method, and between the CT-measured HU and the inner-ellipse method (*p* = 0.093).

## 3. Discussion

Bone density may influence the primary fixation of cementless implants [18], and poor bone density is likely to increase the risk of implant failure. Before deciding on the treatment of TM osteoarthritis, it would therefore be useful to determine the bone density of the trapezium, as a surrogate marker of bone quality. Identification of the cortical perimeter of the trapezium on an anteroposterior (AP) DXA scan is visually challenging, requiring knowledge of the trapezium’s three-dimensional shape and orientation in the wrist, and is therefore time-consuming and error-prone. Furthermore, some overlap between other carpal/metacarpal bones and the trapezium in the scans is unavoidable, whereby bone density from neighboring bones may be included with the trapezium circumference BMD measurement method. This study describes a novel and more simple method, where the BMD of the osteoarthritic trapezium can be determined by measuring the BMD in the center of the trapezium—in the cancellous bone zone within the cortical edges. This is much simpler to mark, and has several clinical advantages. Avoiding the trapezium cortex on the DXA scans eliminates higher BMD values from subchondral sclerosis, cortical bone, and overlap of neighboring bones. In addition, the BMD measured by the inner-ellipse method is likely to be of greater clinical relevance, as it is measured in the center of the trapezium bone where the cup of the TM arthroplasty is fixed. The present study found the two DXA methods—the circumference method and the inner-ellipse method—to have an excellent correlation. 

Substitution of DXA scans with HU measurements from pre-existing CT scans performed for other purposes (such as visualizing the degenerative changes in the TM or neighboring joints) has the advantage of providing an alternative method for determining regional bone quality without additional expense for the hospital or a radiation dose to the patient. However, we only found a moderate correlation between trapezium bone attenuation values (HU units) obtained from CT scans and BMD as measured by DXA. This is in accordance with the findings from a similar study by Schreiber et al. [17] that compared HU units with BMD in the lumbar spine (r^2^ = 0.44, *p* < 0.001).

In the distal radius, we also found a moderate correlation between DXA and HU measurements. Where CT is already used as a pre-operative examination when treating comminuted distal radius fractures, HU may give an indication of the bone quality of the radius and be of value when deciding on the specific fracture treatment e.g., augmentation by external fixation. In a recent study, HU was used to examine bone quality in the trapezium and revealed that bone density of the proximal metacarpal bone and the trapezium was lower in patients with first grade osteoarthritis in the trapeziometacarpal joint as compared to patients with no osteoarthritis [19]. We found 22% lower HU in the trapezium in high grade osteoarthritis patients (HU mean 288) compared with low grade osteoarthritis (HU mean 369) in Schreiber et al. [19]. The low grade osteoarthritis patients from Schreiber et al. had 12% lower HU than control subjects, and our high grade osteoarthritis patients had 31% lower HU then control subjects from Schreiber et al. [19]. However, a limitation may be that OA grading has low intra-rater and inter-rater reliability [20].

BMD was significantly higher in men compared to women with both DXA methods, and also we found a trend in BMD towards a higher BMD in the young age group (mean 49 years) compared with the next age group (mean 59 years). In a comparison with a large European reference data, we found similar BMD of the distal radius for both women and men in the current study.

A major limitation of this study is that only one observer performed the measurements. However, the inner-ellipse method for DXA BMD measurement is technically less difficult than the circumference method, and we postulate that this technique will highly useful for daily clinical use. We have previously tested the inter-observer reliability of trapezium BMD measurement where we found a great correlation of both repeated measurements Concordance correlation coefficient (CCC = 0.96) and repeated scans (CCC = 0.89) [16]. Due to this previous study we did not include inter-observer measurements in this study.

In addition, the HU measurements should be easily applicable to CT scans made for other purposes, and may be considered for an objective judgement of the bone quality when DXA scans are not available. Another limitation of this study is that we did not include a healthy control group. This would be of interest in order to examine the effect of sex and age on BMD measured by DXA and CT.

The influence of bone density and quality on implant survival in TM total joint arthroplasty still remains unclear, and normative data so far have not been presented in larger studies. With the availability of reliable methods for determining the bone density of the trapezium, future research should focus on the relation between implant failure and trapezium bone density.

## 4. Materials and Methods

As part of the standard preoperative evaluation for planned TM total joint arthroplasty, we performed CT and DXA scans of the trapezium on all patients. 

### 4.1. Patients

Sixty-five consecutive patients diagnosed with Eaton–Glickel stage II-IV [21] osteoarthritis of the TM joint were initially included. Six patients had to be excluded due to insufficient CT scanning material, leaving 71 hands (14 male and 57 female) in 59 patients (13 males and 46 females), with a mean age of 59 years; range (40–77).

### 4.2. Image Acquisition and Analysis

**Figure 1:**
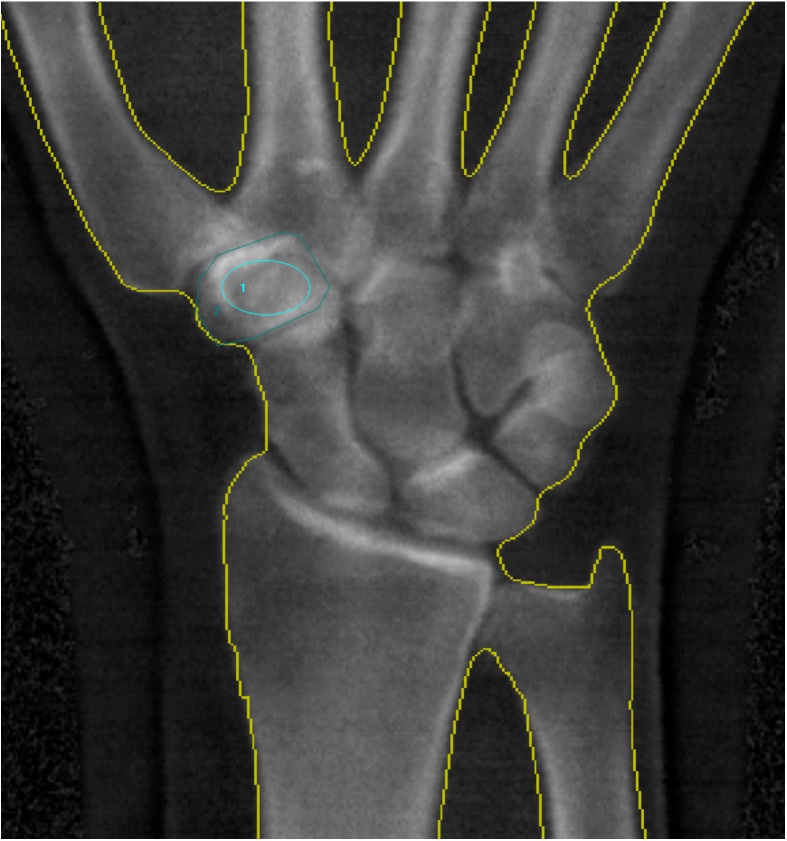
BMD measurement using the inner-ellipse method (1), and the circumference method (2). The BMD is calculated on the basis of the outlined area.

In all patients, we measured the BMD of the trapezium with a GE Lunar iDXA scanner (GE Healthcare, Milwaukee, WI, USA). The hands were placed with the palm against the scanner bed. Image files were processed using the software enCORE version 11.40 (GE Healthcare, Milwaukee, WI, USA) by one observer. In the circumference method, the perimeter of the trapezium was outlined manually, and the BMD calculated based on the outlined area (Figure 1) [16]. In the inner-ellipse method, the BMD in the centre of the trapezium was calculated by placing an ellipse inside the trapezium, avoiding the cortical bone (Figure 1). All measurements were performed by one observer and repeated within eight weeks by the same observer. The BMD and T-score (German reference database) of the distal radius was calculated by the software program, which automatically defined the distal radius very well, but could be corrected manually if needed.

**Figure 2:**
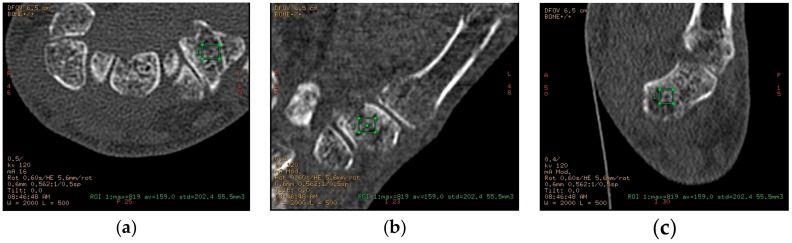
(**a**) Bone mineral density determination by measuring CT attenuation (in HU) in a three-dimensional volume, defined by a rectangular ROI in axial plane; (**b**) Bone mineral density determination by measuring CT attenuation (in HU) in a three-dimensional volume, defined by a rectangular ROI in coronal plane; (**c**) Bone mineral density determination by measuring CT attenuation (in HU) in a three-dimensional volume, defined by a rectangular ROI in sagittal plane. Green square: The region of interest on CT scan which was placed in the cancellous bone of the trapezium in all three (a, b, c) scan planes for assessment of HU values.

The HU measurements were made by one observer using CT scans of the hand and wrist, which had been performed to evaluate osteoarthritic changes in the TM joint (Figure 2). All wrists were CT scanned using a standard bone protocol (0.625 mm slice thickness, bone reconstruction algorithm). 

Most scans were with the wrist in question (left or right) scanned alone, but in a small number of patients both wrists were scanned together in a larger field of view. Axial images were sent to a General Electric Healthcare Advantage workstation, where sagittal and coronal multiplanar reconstructions were performed. Measurements of attenuation values in a three-dimensional (3D) volume of trabecular bone in both trapezium and distal radius were performed on this workstation (Figure 2). 

A three-dimensional region of interest (ROI) was defined by manually drawing a rectangular ROI in all three planes (axial, coronal, sagittal), according to the following constraints: a minimum volume of 50 mm^3^ (trapezium) or 250 mm^3^ (distal radius), no cortical bone was visibly included, and large focal alterations in bone structure were avoided—such as cysts or islands of sclerotic bone. In some cases, such focal changes could not be entirely excluded from the ROI. For each ROI, the following parameters were noted: volume (in mm^3^), maximum attenuation value (in Hounsfield units (HU)), average attenuation (HU), and standard deviation. Each set of measurements (trapezium and distal radius) was performed twice for each wrist by the same observer, with at least 14 days’ (up to 28 days) interval.

### 4.3. Statistical Analysis

We created an age group variable, consisting of three categories from the youngest to oldest with the 24 youngest in the first group (mean age 49.8, 95% CI (47.9–51.8)), the next 23 in the second group (mean age 59.1, 95% CI (58.3–60.0)), and the oldest 24 in the third group (mean age 68.3, 95% CI (66.6–69.9)). These were used to compare the patients’ distal radius BMDs to the reference distal radius BMD values for Northern Europeans [22] within each age group and total by sex using a one-sample *t*-test (Table 1). Test for normality was made using Shapiro-Wilk W test, histograms, and quantile-quantile (QQ) plots. The significance level was set to 0.05. The mean DXA trapezium BMD measurements and CT HU measurement was compared between sex (Table 2) and age groups (Table 3). The effects of sex and age on CT-measured HU and trapezium DXA-measured BMD were tested using multiple regression analysis (Table 4).

The correlation between DXA trapezium BMD inner-ellipse method, DXA trapezium BMD circumference method, and CT HU was tested using Pearson’s correlation test. A significance level of 0.05 was used. We used the Fisher’s z transformation to transform the r-values to z-values, making it possible to calculate 95% confidence intervals (95% CI) to the z-value, which was then transformed back to r-values using Fisher’s reverse transformation. We used the cocor statistical software package [23] to test the difference between the correlations of: (1) CT-measured HU and DXA trapezium inner-ellipse measured BMD and (2) CT-measured HU and DXA trapezium circumference measured BMD.

### 4.4. Reproducibility

We tested the DXA inner-ellipse method intra-observer repeatability comparing the rater’s two measurements on each scan giving two repeatability values. This was further tested by comparing the mean of the two measurements of the first scan to the mean of the two measurements of the second scan.

## 5. Conclusions

The new and technically easier DXA inner-ellipse method can replace the DXA circumference method in estimation of bone density of the trapezium, as there is an excellent correlation between methods. CT scans correlated moderately with DXA measurements in the trapezium and might be useful to examine the bone quality.

Further research should determine the correlation between CT scans and DXA measurements and whether or not poor bone quality may lead to an increased risk of implant failure in trapeziometacarpal total joint replacement.

## Figures and Tables

**Table 1:** Mean BMD from total, ultradistal, and radius 33% measures and the reference mean, reference upper limits for osteopenia and osteoporosis, and *p* value for the difference between data mean and reference mean for Northern Europeans.

Reference Mean And Data Mean	Data Mean	SEM	95% CI	Reference Normal	Reference Osteopenia	Reference Osteoporosis	*p* Value
Total BMD							
Males (N = 14)	0.71	0.02	0.67–0.74	0.74	0.67	0.56	0.06
Females (N = 57)	0.61	0.01	0.58–0.63	0.63	0.57	0.48	0.05
Ultra distal BMD							
Males (N = 14)	0.50	0.01	0.47–0.52	0.51	0.46	0.38	0.22
Females (N = 57)	0.42	0.01	0.39–0.44	0.43	0.38	0.32	0.25
Radius 33% BMD							
Males (N = 14)	0.94	0.02	0.90–0.98	0.96	0.86	0.71	0.36
Females (N = 57)	0.81	0.01	0.78–0.83	0.81	0.72	0.59	0.74

SEM, Standard error of the mean; Reference Osteopenia, Upper BMD limit for reference osteopenia (Reference mean—(1 × SD)); Reference Osteoporosis, Upper BMD limit for reference osteoporosis (Reference mean—(2.5 × SD)); *p* value, *p* value for the difference between data mean and reference normal.

**Table 2:** Means from the two DXA methods and the CT method by sex.

DXA And CT Mean	Mean	SEM	95% CI
DXA circumference (g/cm^2^)			
Male hands (n = 14)	0.78	0.05	0.68–0.89
Female hands (n = 57)	0.65	0.02	0.62–0.69
DXA inner-ellipse (g/cm^2^)			
Male hands (n = 14)	0.73	0.05	0.63–0.83
Female hands (n = 57)	0.61	0.02	0.57–0.64
CT scans (HU)			
Male hands (n = 14)	277.43	17.89	241.75–313.11
Female hands (n = 57)	298.44	11.89	274.72–322.15

SEM, Standard error of the mean.

**Table 3:** Means from the two DXA methods and CT method by age group.

DXA and CT Mean	Mean	SEM	95% CI
DXA circumference (g/cm^2^)			
Age group 1 (N = 24)	0.72	0.03	0.66–0.77
Age group 2 (N = 23)	0.64	0.03	0.59–0.69
Age group 3 (N = 24)	0.69	0.04	0.62–0.77
DXA inner-ellipse (g/cm^2^)			
Age group 1 (N = 24)	0.67	0.03	0.62–0.77
Age group 2 (N = 23)	0.59	0.03	0.53–0.64
Age group 3 (N = 24)	0.64	0.04	0.56–0.71
CT scans (HU)			
Age group 1 (N = 24)	310.71	17.72	275.34–346.04
Age group 2 (N = 23)	309.72	20.62	268.59–350.85
Age group 3 (N = 24)	263.10	12.83	237.52–288.69

SEM, Standard error of the mean.

**Table 4:** Multiple regression analysis showing the effects of sex and age on CT measured HU and DXA measured BMD by the circumference and inner-ellipse method respectively.

Variable	Coef.	SEM	t	*p* > [t]	95% CI
DXA circumference (g/cm^2^)					
Sex	−0.13	0.04	−3.22	0.002	−0.22–−0.05
Age	−0.00	0.00	−1.65	0.103	−0.01–0.00
DXA inner-ellipse (g/cm^2^)					
Sex	−0.13	0.04	−3.00	0.004	−2.11–−0.04
Age	−0.00	0.00	−1.79	0.078	−0.01–0.00
CT scans (HU)					
Sex	17.24	25.26	0.68	0.497	−33.16–67.65
Age	−2.23	1.19	−1.87	0.065	−4.60–0.14

SEM, Standard error of the mean. Note: Bold *p* value denotes significance.

**Table 5:** Repeatability measurements (intra-observer) of trapezium BMD measurements by the inner-ellipse method and the CT HU trapezium measurement.

Measurement	Mean (95% CI), g/cm^3^	SD_dif_	Bias (LOA), g/cm^3^	*p* Value	ICC
Intra-observer repeated measurements					
DXA Scan 1 (N = 71)	0.62 (0.58–0.65)	0.058	−0.007 (0.114)	0.00	0.93
DXA Scan 2 (N = 71)	0.64 (0.61–0.62)	0.061	0.003 (0.120)	0.00	0.92
CT Scan (N = 71)	294.30 (274.0–314.6)	47.88	18.79 (93.84)	0.00	0.84

SDdif, Random variation of double measurements or scans; Bias, Systematic variation between two measurements or two scans; LOA: Limits of agreement around the bias (SDdif * 1.96); Intra-observer repeated measurement, trapezium BMD, and HU measured twice on the same scan.
